# Myosteatosis and the clinical outcomes of patients with liver cirrhosis: A meta-analysis

**DOI:** 10.1371/journal.pone.0310017

**Published:** 2024-09-12

**Authors:** Haojie Xue, Yihan Liu, Yang Liu, Han Li, Qian Liang, Longhui Ma, Junying Liu, Ming Zhao

**Affiliations:** 1 Zhoukou Central Hospital Affiliated to Xinxiang Medical University, Zhoukou City, Henan Province, China; 2 Ward 1, Department of Gastroenterology, Zhoukou Central Hospital, Zhoukou City, Henan Province, China; 3 Zhoukou Central Hospital, Zhoukou City, Henan Province, China; Ankara University, TÜRKIYE

## Abstract

**Objectives:**

This study aimed to examine the potential correlation between myosteatosis and the prognosis of patients diagnosed with liver cirrhosis by a meta-analysis.

**Methods:**

Cohort studies of relevance were acquired through comprehensive searches of the Medline, Web of Science, and Embase databases. To account for heterogeneity, a random-effects model was employed to combine the findings.

**Results:**

The meta-analysis included 10 retrospective and four prospective cohort studies, encompassing a total of 4287 patients diagnosed with cirrhosis. The pooled findings indicated a notable decline in transplant-free survival (TFS) among individuals with liver cirrhosis and myosteatosis compared to those without this condition (risk ratio: 1.94; 95% confidence interval: 1.61 to 2.34, *p* < 0.001; I^2^ = 49%). The predefined subgroup analyses demonstrated consistent findings across various categories, including Asian and non-Asian studies, prospective and retrospective cohort studies, patients with cirrhosis overall and those who underwent transjugular intrahepatic portosystemic shunt, studies with different follow-up durations (< or ≥ 24 months), studies employing univariate and multivariate analyses, and studies with and without an adjustment for sarcopenia (*p* > 0.05 for all subgroup differences). Additionally, Egger’s regression test indicated the presence of significant publication bias (*p* = 0.044). However, trim-and-fill analysis by including three hypothesized studies showed consistent results.

**Conclusions:**

The presence of myosteatosis in individuals diagnosed with liver cirrhosis may potentially be linked to a poor TFS prognosis. Further investigations are required to ascertain whether enhancing myosteatosis could potentially yield a survival advantage for this particular patient population.

## 1. Introduction

Liver cirrhosis is a chronic liver disease characterized by irreversible fibrosis and a progressive loss of liver function [1]. It can result from various etiologies, including chronic hepatitis B and C infection, excessive alcohol consumption, and non-alcoholic fatty liver disease [2]. The progression of cirrhosis can lead to various complications, such as portal hypertension, hepatic encephalopathy, variceal bleeding, and hepatocellular carcinoma, significantly impairing patients’ quality of life and increasing mortality rates [3,4]. Understanding these impacts is crucial for managing and improving the outcomes in patients with cirrhosis.

Pathological skeletal muscle disorders are commonly observed in individuals who have been diagnosed with liver cirrhosis [5,6]. A notable skeletal muscle disorder in cirrhosis is sarcopenia, which is characterized by a widespread decrease in both muscle volume and function [7,8]. In addition to sarcopenia, recent research has emphasized the significance of another skeletal muscle disorder in liver cirrhosis patients, namely myosteatosis [9]. Defined as pathological skeletal muscle fat infiltration, myosteatosis may be observed in up to 50% of patients with cirrhosis [9,10]. Factors contributing to myosteatosis include systemic inflammation, hormonal imbalances, malnutrition, and physical inactivity [9,11]. Systemic inflammation promotes lipolysis and impairs muscle protein synthesis, while hormonal imbalances, such as reduced levels of testosterone and insulin-like growth factor 1, exacerbate muscle wasting and fat infiltration [12]. Malnutrition and physical inactivity further contribute to the loss of muscle mass and function [12]. Furthermore, the differentiation of muscle stem cells into adipocytes due to aging may also play a role in the progression of myosteatosis [9]. Various diagnostic methods can be used to assess muscle fat infiltration and overall muscle health; in particular, imaging techniques, such as computed tomography (CT), magnetic resonance imaging, and dual-energy X-ray absorptiometry [13]. Although there is no universally accepted definition of myosteatosis, CT is the most common imaging technique used for evaluating myosteatosis because it can directly detect fat infiltration within muscles [13].

Despite some preliminary clinical observations suggesting a correlation between myosteatosis and a higher occurrence of cirrhosis complications and higher mortality rates [14–16], there has been a lack of comprehensive evaluation through meta-analysis regarding the impact of myosteatosis on the clinical prognosis of cirrhosis patients. Furthermore, it remains unclear whether the potential prognostic effectiveness of myosteatosis in cirrhosis patients is consistent in those who have undergone a transjugular intrahepatic portosystemic shunt (TIPS) procedure [17]. Moreover, considering the high occurrence of sarcopenia and myosteatosis in individuals with advanced liver diseases [18], it is crucial to determine whether the prognostic significance of myosteatosis in cirrhotic patients remains unaltered in the presence of sarcopenia. In view of these knowledge gaps, we undertook this comprehensive review and meta-analysis to investigate the association between myosteatosis and the prognosis of individuals diagnosed with liver cirrhosis.

## 2. Materials and methods

The current meta-analysis followed the guidelines specified in the Preferred Reporting Items for Systematic Reviews and Meta-Analyses statement (PRISMA 2020) [19] **(S1 Checklist)** and the Cochrane Handbook for Systematic Reviews and Meta-analyses [20] throughout all stages of the study, including the study design, data collection, statistical analysis, and interpretation of the findings. The protocol for the manuscript has been registered in PROSPERO with the registration code CRD42024573323. Institutional Review Board approval was not required because this is a meta-analysis.

### 2.1. Literature search

To identify studies relevant to the aim of the meta-analysis, we searched the Medline, Web of Science, and Embase databases utilizing comprehensive search terms involving ("myosteatosis" OR "muscle attenuation" OR "muscle density" OR "intramuscular adipose tissue infiltration" OR "intramuscular adipose tissue content" OR "intramuscular adipose tissue deposition" OR "intramuscular fat content" OR "intramuscular fat infiltration" OR "intramuscular fat deposition") AND ("cirrhosis" OR "cirrhotic" OR "liver fibrosis" OR "liver" OR "hepatic" OR "hepatitis) AND ("prognosis" OR "survival" OR "mortality" OR "death" OR "deaths" OR "transplant" OR "transplantation" OR "transplant-free survival" OR "TFS"). The search was restricted to human studies, specifically focusing on full-length articles published in peer-reviewed journals in the English language. Since the gray literature, such as conference abstracts and unpublished data, generally does not undergo peer review processes, including such data in the meta-analysis could affect the reliability of the meta-analysis results; hence the gray literature was not included in this meta-analysis. Additionally, the references of relevant original and review articles were manually examined to identify other potentially pertinent studies. The literature encompassing the period from the establishment of the databases up to August 13, 2024 was thus thoroughly screened.

### 2.2. Inclusion and exclusion criteria

The eligibility criteria for the potentially included studies encompassed the following aspects: (1) the studies had to be observational in nature and published as full-length articles; (2) the participants had to be adult individuals aged 18 years old or older diagnosed with liver cirrhosis; (3) myosteatosis was evaluated at the beginning of each study, and the definition of myosteatosis was consistent with that used in all the included studies; and (4) the studies reported the disparity in transplant-free survival (TFS) between patients with cirrhosis with and without myosteatosis at baseline.

The exclusion criteria were: (1) studies in patients with hepatocellular carcinoma (HCC) only; (2) studies including patients after liver transplant; (3) studies that did not evaluate the status of myosteatosis or did not report the outcome of interest; or (4) preclinical studies, reviews, or editorials. Studies with insufficient outcome data were excluded, and the analysis was only for available data. If studies with overlapping populations were retrieved, the one with the largest sample size was included for the meta-analysis.

### 2.3. Study quality evaluation and data extraction

The process of conducting the literature search, identifying relevant studies, evaluating their quality, and collecting data was performed independently by two authors (HX and YL). In cases of discrepancy, the reviewers (HX and YL) discussed the differences with an aim to reach consensus. If consensus could not be reached, a third reviewer (JL) was consulted to resolve the disagreement. The quality assessment of the included studies was conducted using the Newcastle–Ottawa Scale (NOS) [21], which evaluates three dimensions: the selection of the cases and controls, comparability between groups, and the measurement of exposure. Each study was awarded a maximum of nine stars. Studies with NOS scores of 7 or higher were considered high quality, while those with scores of 4–6 were deemed moderate quality, and those with scores below 4 were considered low quality [21]. The final data extraction was performed on August 13, 2024 by HX and YL. The collected data from each study comprised diverse components for subsequent analysis, including details about the study (such as author, year, country, and design), participant characteristics (such as sample size, age, sex, and etiology of liver diseases), specifics of myosteatosis measurement (such as methods, cutoff values, and number of patients with myosteatosis at baseline), follow-up durations, and variables adjusted when reporting the association between myosteatosis and TFS in patients with cirrhosis. Missing data of study or patient characteristics were presented as not reported (NR) in the data extraction table, and studies with missing outcome data were excluded from the meta-analysis.

### 2.4. Statistics

The present study aimed to evaluate the association between myosteatosis and TFS in patients with cirrhosis by calculating risk ratios (RRs) and corresponding 95% confidence intervals (CIs). RRs and standard errors (SEs) were determined using either 95% CIs or *p*-values, and a logarithmical transformation was applied to stabilize and normalize the variance. To assess study heterogeneity, the Cochrane Q test and I^2^ statistics were utilized, with an I^2^ value exceeding 50% indicating significant heterogeneity [22]. A random-effects model was employed to combine the results, considering the potential influence of heterogeneity [20]. Sensitivity analyses were performed by systematically excluding individual studies to further investigate the results. The study also conducted predefined subgroup analyses to evaluate how the characteristics of the studies may have influenced the outcomes, such as the cutoff for the diagnosis of myosteatosis, the study country, design, use of TIPS, follow-up duration, analytic model (univariate or multivariate), and adjustment for sarcopenia. For instance, differentiating by body mass index (BMI) in diagnosing myosteatosis helps assess if the diagnostic criteria may affect the outcomes, comparing Asian and non-Asian studies allows exploring regional or genetic influences, examining prospective versus retrospective studies helps understand how the study design impacts the findings, analyzing overall cirrhosis patients versus those who have undergone TIPS evaluates if the disease stage or interventions may alter the results, considering the follow-up duration differences provides insights into short-term versus long-term effects, univariate versus multivariate analyses reveal if associations hold when adjusting for other factors, and comparing studies with and without sarcopenia adjustment allows clarifying whether myosteatosis impacts the outcomes independently or in conjunction with sarcopenia. The subgroups were defined based on the medians of the continuous variables. To assess publication bias in the meta-analysis, funnel plots were created and visually assessed for symmetry, in addition to conducting an Egger’s regression test [23]. If significant publication bias was detected, a trim-and-fill analysis was employed [24]. The current analysis incorporated hypothesized studies to achieve funnel plot symmetry and determine if the results of the meta-analysis remained consistent when including these hypothesized studies [24]. Statistical analysis was performed using RevMan (Version 5.1; Cochrane Collaboration, Oxford, UK) and Stata software (version 12.0; Stata Corporation, College Station, TX, USA). A two-sided *p*-value of less than 0.05 was considered statistically significant.

## 3. Results

### 3.1. Study inclusion

**Fig 1:**
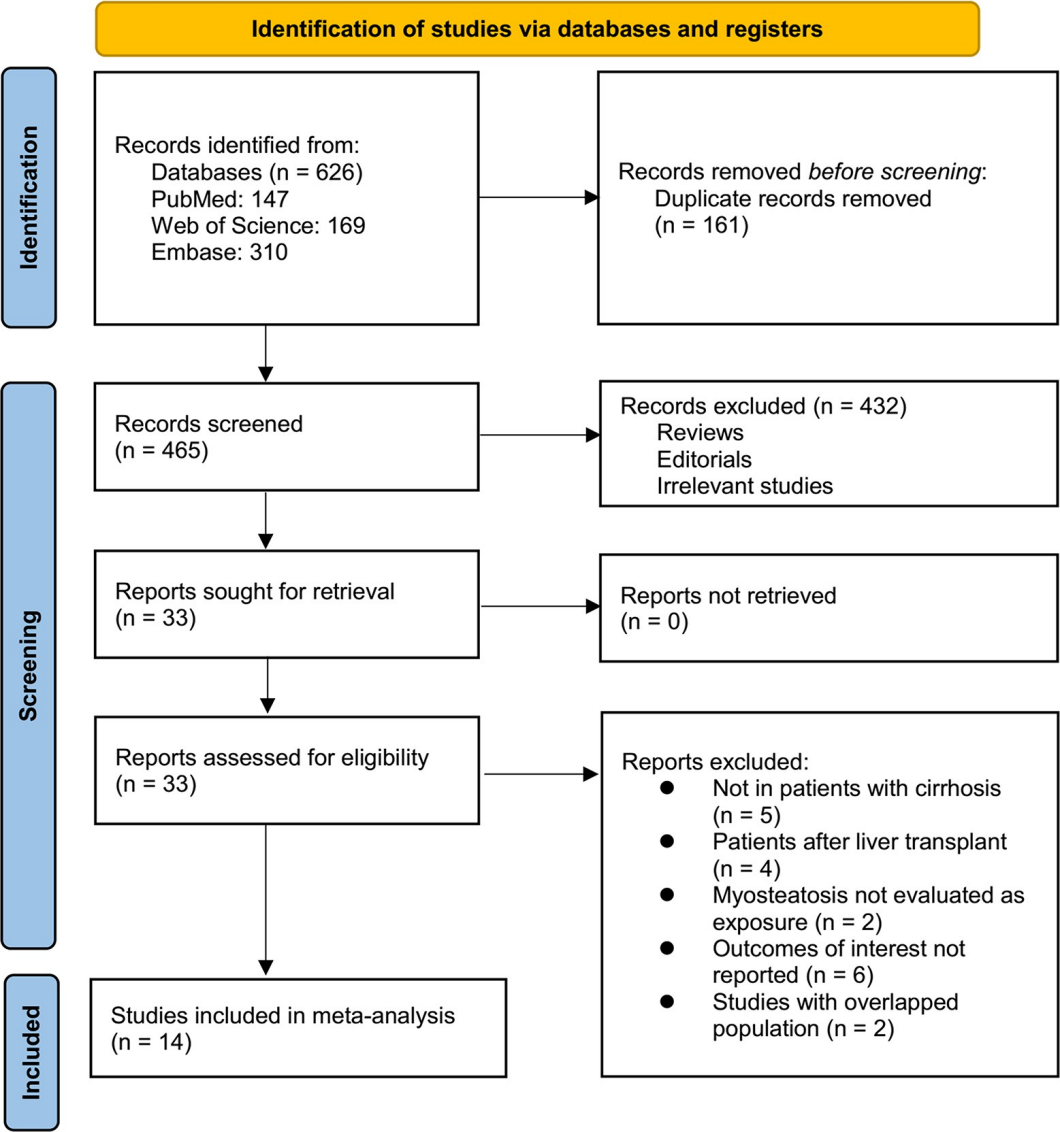
Flowchart showing the database search and study inclusion process.

The process for identifying relevant studies for inclusion in the meta-analysis is presented in **Fig 1**. In brief, 626 potentially relevant records were obtained after comprehensive searches of the three aforementioned databases, and 161 of these were immediately excluded due to duplication. Subsequently, screening the remaining records via considering their titles and abstracts led to the further exclusion of 432 more studies, mostly because they were not related to the aim of the meta-analysis. Of the 33 remaining records, their full texts were read by two independent authors in a further screening round, and 19 of these were removed for various reasons, as listed in **Fig 1**. Finally, 14 cohort studies remained and were considered to be suitable for the subsequent quantitative analyses [16,25–37]. A list of excluded studies along with their justifications is provided in **S1 Table**.

### 3.2. Overview of the studies’ characteristics

**Table 1** presents the summarized characteristics of the included studies. Overall, 14 cohort studies, comprising 4 prospective cohort studies [16,27,34,37] and 10 retrospective cohort studies [25,26,28–33,35,36], were included in the meta-analysis. These studies were published between 2016 and 2024, and performed in Canada, Greece, Italy, the United States, and China. Eleven of them included patients with overall liver cirrhosis [16,25–27,29–32,34,36,37], and three of them included patients with cirrhosis after undergoing TIPS [28,33,35]. Overall, 4287 patients with cirrhosis were included, with mean ages from 52 to 63 years old across the studies, and the proportions of men varied from 53% to 78%. The mean model for end-stage liver disease (MELD) scores at baseline were 11 to 15, and the mean Child–Pugh scores at baseline were 7.0 to 9.3. The status of myosteatosis was evaluated at the third or fourth lumbar level using CT, and the cutoffs for defining myosteatosis varied among the studies. The cutoffs for defining myosteatosis were BMI-differentiated in seven of the studies [16,25,27,32,34,35,37], but not in the other seven studies [26,28–31,33,36]. Accordingly, 1706 (40%) patients had myosteatosis at baseline. The follow-up durations ranged from 12 to 45 months. Univariate analyses were used in eight studies [16,26,28,30,32,33,35,37] when the association between myosteatosis and TFS was investigated. In the other six studies [25,27,29,31,34,36], multivariate analyses were used and potential confounding factors were adjusted, such as age, sex, MELD score, complications of cirrhosis, and sarcopenia, to varying degrees. The NOS of the included studies ranged from six to nine stars, and 11 of the 14 studies were of good quality according to these scores (**Table 2**).

**Table 1:** Characteristics of the included cohort studies.

Study	Country	Design	Diagnosis	Viral etiology (%)	Alcoholic etiology (%)	Patient number	Mean age (years)	Male (%)	Mean MELD score	Mean CPS	Methods for myosteatosis measurement	Number of patients with myosteatosis	Definition of myosteatosis	Median follow-up duration (months)	Variables adjusted
Montano 2016 [25]	Canada	RC	Cirrhosis	46	23	678	57	67	15	9.3	L3, CT	353	< 41 HU for patients with BMI < 25, and < 33 for patients with BMI ≥ 25	21	Age, sodium, sarcopenia, MELD score, CPS
Kalafateli 2018 [26]	Greece	RC	Cirrhosis	23	46	98	63	72	11	7	L4, CT	20	< 35 HU (lowest quintile)	45	None
Lattanzi 2019 [27]	Italy	PC	Cirrhosis	24	57	249	60	76	14	NR	L3, CT	135	< 41 HU for patients with BMI < 25, and < 33 for patients with BMI ≥ 25	15	Age, sex, etiology, MELD score, complications, and sarcopenia
Nardelli 2019 [16]	Italy	PC	Cirrhosis	70	20	64	57	75	14	7.7	L3, CT	24	< 41 HU for patients with BMI < 25, and < 33 for patients with BMI ≥ 25	16	None
Shoreibah 2019 [28]	USA	RC	Cirrhosis after TIPS	35	33	241	56	62	12	NR	L3, CT	78	< 29.4 HU (ROC analysis derived)	30	None
Hou 2021 [29]	China	RC	Cirrhosis	24	21	274	62	53	12	NR	L3, CT	48	IMAC (male, −0.44; female, −0.37)	36	Age, sex, etiology, MELD score, Child–Pugh class, and sarcopenia
Chen 2022 [30]	China	RC	Cirrhosis	57	16	223	52	65	12	NR	L3, CT	111	< 39.8 HU in men, < 30.7 HU in women (ROC analysis derived)	36	None
Ebadi 2022 [31]	Canada	RC	Cirrhosis	45	25	855	56	63	15	NR	L3, CT	285	< 33 HU in men, < 28 HU in women (lowest tertile)	24	Age, sex, etiology, albumin, MELD score, complications, BMI, and sarcopenia
Nardelli 2022 [32]	Italy	RC	Cirrhosis	54	11	114	58	76	13	7.6	L3, CT	57	< 41 HU for patients with BMI < 25, and < 33 for patients with BMI ≥ 25	14	None
Zhang 2022 [33]	China	RC	Cirrhosis after TIPS	55	19	273	54	71	11	7	L3, CT	102	< 49.9 HU (ROC analysis derived)	12	None
Zeng 2023 [36]	China	RC	Cirrhosis	44	13	480	55	62	12	7.4	L3, CT	147	< 38.9 HU in men, < 32.8 HU in women	24	Age, sex, albumin, complications, and sarcopenia
Geladari 2023 [34]	Greece	PC	Cirrhosis	47	22	197	61	67	11	7	L3, CT	145	< 41 HU for patients with BMI < 25, and < 33 for patients with BMI ≥ 25	12	Age, sex, BMI, MELD score, physical performance, and sarcopenia
Yin 2023 [35]	China	RC	Cirrhosis after TIPS	69	16	108	53	78	11	7.4	L3, CT	35	< 41 HU for patients with BMI < 25, and < 33 for patients with BMI ≥ 25	24	None
Di Cola 2024 [37]	Italy	PC	Cirrhosis	20	40	433	57	71	13	7.5	L3, CT	166	< 41 HU for patients with BMI < 25, and < 33 for patients with BMI ≥ 25	12	None

MELD, model for end-stage liver disease; RC, retrospective cohort; PC, prospective cohort; TIPS, transjugular intrahepatic portosystemic shunt; NR, not reported; L3, third lumbar; CT, computed tomography; BMI, body mass index; CPS, Child–Pugh score.

**Table 2:** Study quality evaluation via the Newcastle–Ottawa Scale.

Study	Representativeness of the exposed cohort	Selection of the non-exposed cohort	Ascertainment of exposure	Outcome not present at baseline	Control for age and sex	Control for other confounding factors	Assessment of outcome	Sufficiently long follow-up duration	Adequacy of follow-up of the cohorts	Total
Montano 2016 [25]	0	1	1	1	1	1	1	1	1	8
Kalafateli 2018 [26]	1	1	1	1	0	0	1	1	1	7
Lattanzi 2019 [27]	1	1	1	1	1	1	1	1	1	9
Nardelli 2019 [16]	1	1	1	1	0	0	1	1	1	7
Shoreibah 2019 [28]	0	1	1	1	0	0	1	1	1	6
Hou 2021 [29]	1	1	1	1	1	1	1	1	1	9
Chen 2022 [30]	1	1	1	1	0	0	1	1	1	7
Ebadi 2022 [31]	1	1	1	1	1	1	1	1	1	9
Nardelli 2022 [32]	1	1	1	1	0	0	1	1	1	7
Zhang 2022 [33]	0	1	1	1	0	0	1	1	1	6
Zeng 2023 [36]	0	1	1	1	1	1	1	1	1	8
Geladari 2023 [34]	1	1	1	1	1	1	1	1	1	9
Yin 2023 [35]	0	1	1	1	0	0	1	1	1	6
Di Cola 2024 [37]	1	1	1	1	0	0	1	1	1	7

### 3.3. Results of the meta-analysis

**Fig 2:**
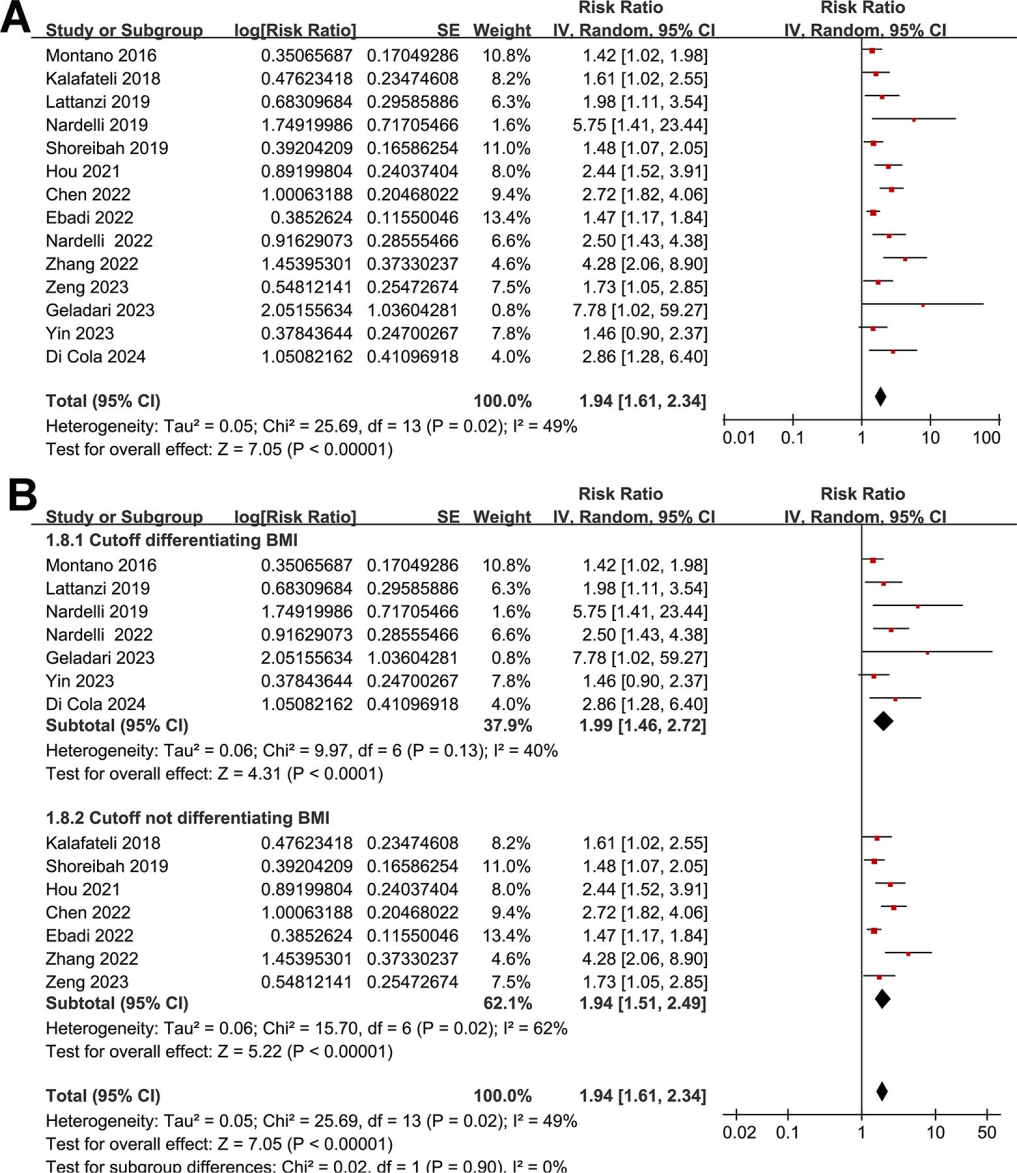
Forest plots for the meta-analysis of the association between myosteatosis and the TFS of patients with liver cirrhosis: A, overall meta-analysis; and B, subgroup analysis according to whether BMI was differentiated when designating the cutoff for the diagnosis of myosteatosis.

**Fig 3:**
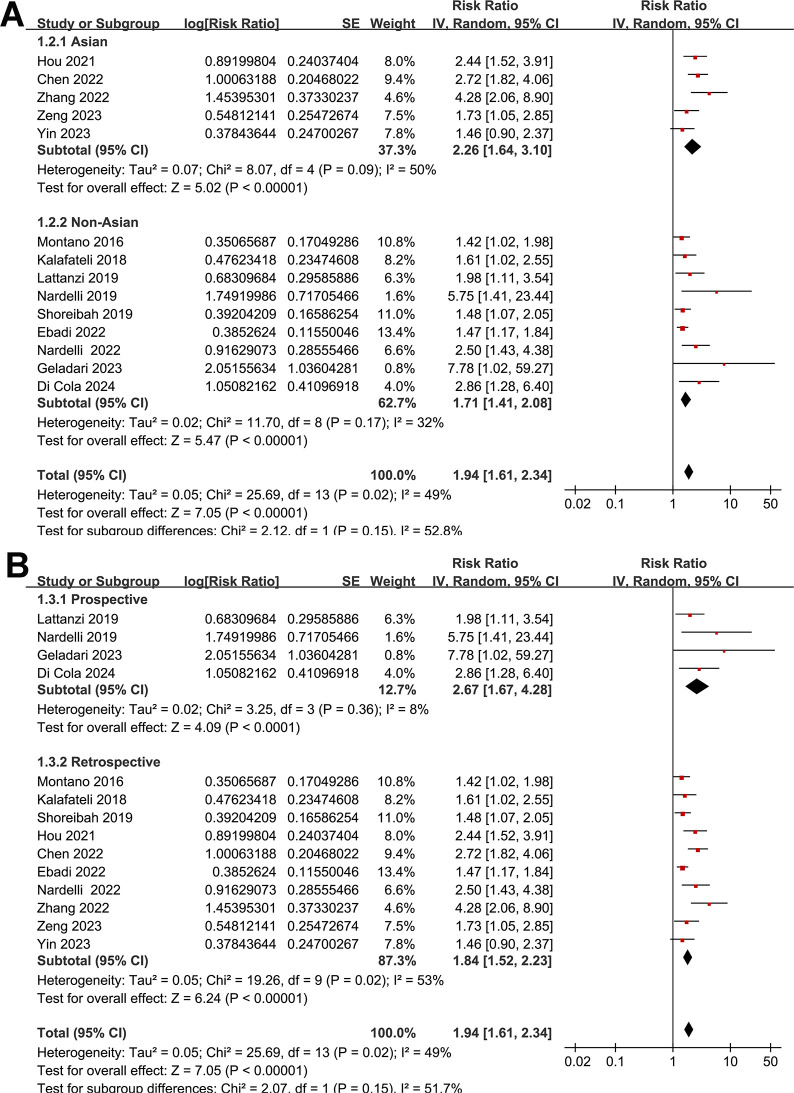
Forest plots for the subgroup analyses of the association between myosteatosis and the TFS of patients with liver cirrhosis: A, subgroup analysis according to the study country; and B, subgroup analysis according to the study design.

**Fig 4:**
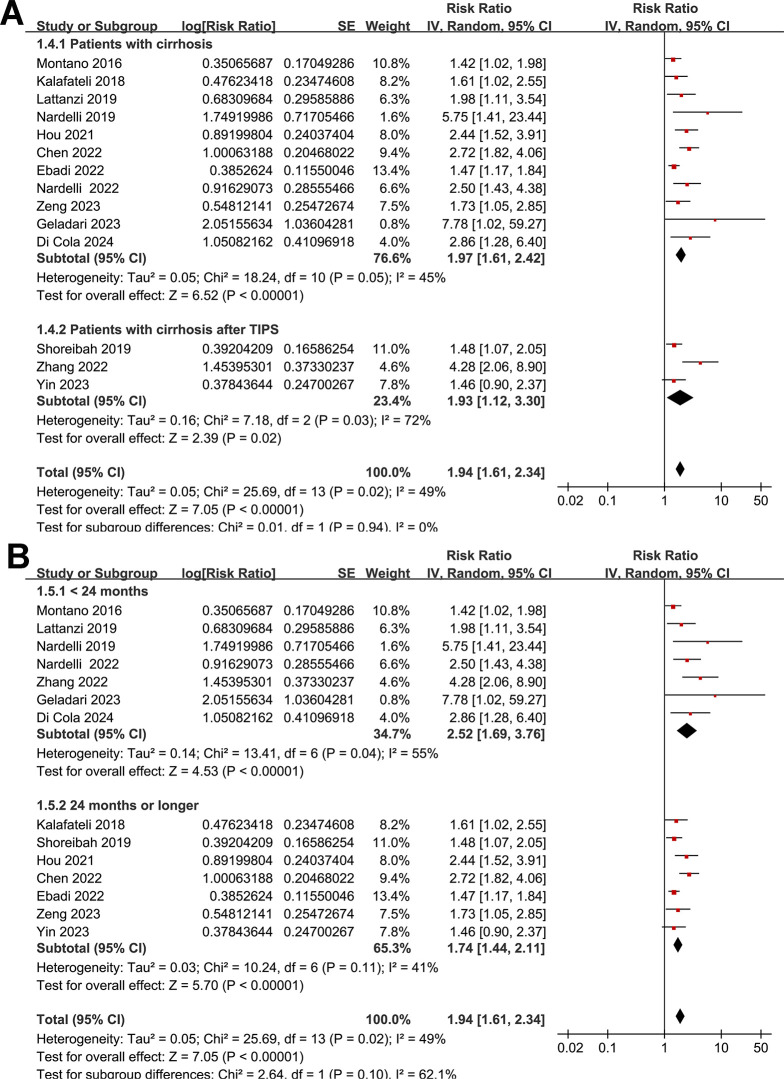
Forest plots for the subgroup analyses of the association between myosteatosis and the TFS of patients with liver cirrhosis: A, subgroup analysis according to treatment by TIPS; and B, subgroup analysis according to the follow-up duration.

**Fig 5:**
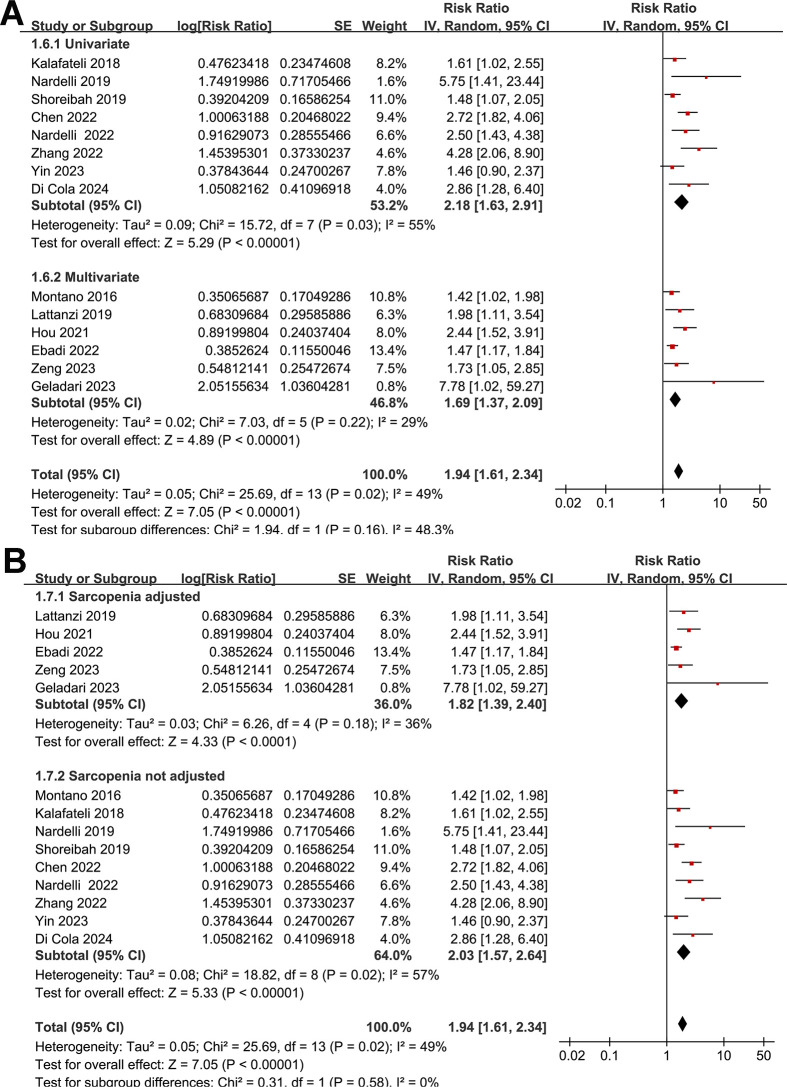
Forest plots for the subgroup analyses of the association between myosteatosis and the TFS of patients with liver cirrhosis: A, subgroup analysis according to the analytic models; and B, subgroup analysis according to the adjustment for sarcopenia.

None of the included studies reported the SEs and so the SEs were backtransformed from 95% CIs in all the included studies. Also, none of the included studies reported the *p*-value only. Pooling the results with a random-effects model showed that compared to those without myosteatosis, patients with liver cirrhosis and myosteatosis had a poor TFS (RR: 1.94, 95% CI: 1.61 to 2.34, *p* < 0.001; I^2^ = 49%; **Fig 2A**). Further sensitivity analyses by excluding one study at a time showed consistent results (RR: 1.84 to 2.03, *p* all < 0.05). Predefined subgroup analyses showed similar results in studies with cutoffs for the diagnosis of myosteatosis with and without a differentiation based on BMI (*p* for subgroup difference = 0.90, **Fig 2B**), in Asian and non-Asian studies (*p* for subgroup difference = 0.15, **Fig 3A**), in the prospective and retrospective cohort studies (*p* for subgroup difference = 0.15, **Fig 3B**), in the overall patients with cirrhosis and in the patients after TIPS (*p* for subgroup difference = 0.94, **Fig 4A**), in studies with a follow-up duration < or ≥ 24 months (*p* for subgroup difference = 0.10, **Fig 4B**), in studies with univariate and multivariate analyses (*p* for subgroup difference = 0.16, **Fig 5A**), and in studies with and without an adjustment for sarcopenia (*p* for subgroup difference = 0.58, **Fig 5B**).

### 3.4. Publication bias evaluation

**Fig 6:**
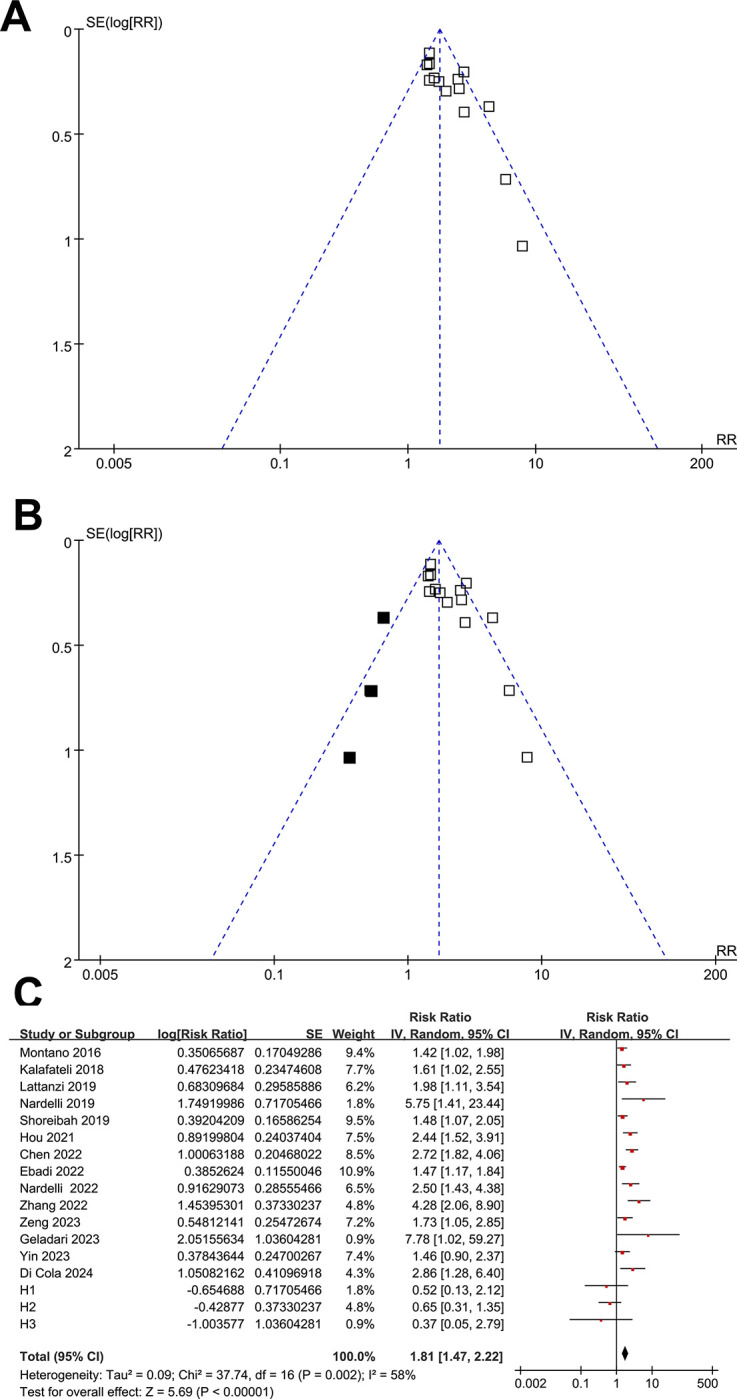
Funnel plots with trim-and-fill analysis for evaluating the possible publication bias of the meta-analysis of the association between myosteatosis and the TFS of patients with liver cirrhosis: A, original funnel plots showing asymmetry; B, funnel plots when incorporating the three hypothesized studies (black square) showing symmetry; C, meta-analysis incorporating the three hypothesized studies (H1, H2, and H3), showing consistent results.

The asymmetrical nature of the funnel plots observed during the meta-analysis investigating the relationship between myosteatosis and TFS in patients with cirrhosis suggested the presence of publication bias (**Fig 6A**). This suspicion was further supported by the significant findings of the Egger’s regression test (*p* = 0.044). To address this bias, a trim-and-fill analysis was conducted, resulting in the inclusion of three hypothesized studies, which allowed the generation of symmetrical funnel plots (**Fig 6B**). The subsequent meta-analysis, incorporating these three hypothesized studies, yielded consistent results (RR: 1.81, 95% CI: 1.47 to 2.22, *p* < 0.001; I^2^ = 58%; **Fig 6C**).

## 4. Discussion

This study conducted a systematic review and meta-analysis, incorporating data from 14 cohort studies, to examine the correlation between myosteatosis and liver cirrhosis. The results indicated that individuals with liver cirrhosis and myosteatosis experienced a notably reduced TFS over a follow-up period of up to 45 months, in comparison to those without myosteatosis. Additionally, the sensitivity analysis, which involved the exclusion of one study at a time, consistently supported these findings. Furthermore, subgroup analyses indicated that the correlation between myosteatosis and an unfavorable TFS in individuals with liver cirrhosis remained unaffected across the various subgroups. Despite the indication of potential publication bias, the utilization of trim-and-fill analysis, which involved the inclusion of three imputed hypothetical studies, did not yield significant alterations to the outcomes of the meta-analysis. In conclusion, the combined outcomes of this meta-analysis provide evidence of a correlation between myosteatosis and an unfavorable TFS in individuals diagnosed with liver cirrhosis.

During the preparation of the meta-analysis, another systematic review and meta-analysis was published evaluating the prevalence and impact on the outcome of myosteatosis in patients with cirrhosis [10]. This systematic review is different from ours because it mainly focused on the prevalence of myosteatosis in patients with cirrhosis (cross-sectional studies were mostly included) [10], while ours was mainly focused on the influence of myosteatosis on the TFS of patients with cirrhosis (cohort studies included only). Although mortality rates were also compared between patients with and without myosteatosis in that recently published meta-analysis, the results were based on crude data only, which may be influenced by confounding factors. From this perspective, it is crucial to recognize the methodology employed in the present meta-analysis before interpreting the results. Notably, a thorough search of three widely utilized electronic databases was conducted, resulting in the identification of three contemporary cohort studies that align with the objectives of this meta-analysis. Nine out of the 14 cohort studies included in this analysis were published within the past three years, thus offering up-to-date insights into the role of myosteatosis in patients with cirrhosis. Furthermore, only cohort studies were considered, allowing for the examination of a longitudinal relationship between myosteatosis and a poor TFS in cirrhosis. Additionally, the robustness of the findings was further confirmed through various sensitivity and subgroup analyses. Notably, our subgroup analysis results indicated that the association between myosteatosis and a poor TFS in cirrhosis was not limited to patients with overall cirrhosis, but also to those after the treatment of TIPS. This is important because TIPS has been shown to prevent decompensation and improve the survival of patients with cirrhosis [17]. Moreover, a previous study confirmed that TIPS creation could strongly prevent psoas muscle attenuation, which was found to be independently associated with a reduced mortality risk in these patients [38]. Furthermore, subgroup analysis demonstrated consistent outcomes in studies employing both univariate and multivariate analyses, indicating that the correlation between myosteatosis and an unfavorable TFS might be unaffected by potential confounding variables, such as age, sex, and the baseline MELD score. These consistent findings underscore the reliability of myosteatosis as a prognostic factor for a poor TFS in patients with cirrhosis.

The potential mechanisms underlying the correlation between myosteatosis and poor TFS in individuals diagnosed with liver cirrhosis are likely to be multifactorial. Myosteatosis has been linked to an increased susceptibility to various complications in cirrhotic patients, including a higher incidence of HCC [14] and hepatic encephalopathy (HE) [39]. The occurrence of these complications can further detrimentally impact the prognosis of affected individuals. Furthermore, a recent investigation conducted on patients with end-stage liver disease demonstrated a significant association between myosteatosis and reduced cardiorespiratory fitness [15], which may also have substantially contributed to the aforementioned decline in TFS. It is noteworthy that multiple studies have posited a potential correlation between diminished fat accumulation in the skeletal muscle of individuals with cirrhosis and a decreased likelihood of complications, such as HE [40], as well as improved survival rates [38,41]. These findings imply that myosteatosis could potentially serve as a therapeutic focus for patients with cirrhosis. Pathophysiologically, myosteatosis, characterized by excessive fat infiltration in skeletal muscles, is thought to impair muscle function and metabolic health [42]. Studies have demonstrated that fat infiltration in muscles is associated with increased systemic inflammation and insulin resistance, which are known to negatively impact the prognosis in various diseases [43,44]. Additionally, metabolic dysfunction associated with myosteatosis can contribute to diminished muscle mass and strength, further affecting the overall health and survival outcomes of patients with cirrhosis [9]. These insights highlight the importance of addressing myosteatosis in the management and prognosis of patients with cirrhosis. However, to the best of our knowledge, the exact molecular signaling pathways linking myosteatosis and poor prognosis for cirrhosis remain to be determined and more studies are warranted in the future to address the gaps in knowledge.

This study has some limitations to note. One important issue to highlight is that the diagnostic cutoff for myosteatosis varied among the included studies. Different thresholds for diagnosing myosteatosis can lead to variability in patient classification, which may affect the observed associations between myosteatosis and clinical outcomes. However, as far as we know, a universal definition of myosteatosis is yet to be established. Furthermore, the majority of the incorporated studies exhibited a retrospective design, thereby potentially subjecting the outcomes of the meta-analysis to recall and selection biases. To substantiate the findings of the meta-analysis, it is imperative to conduct extensive prospective cohort studies on a large scale. Another limitation is the potential influence of heterogeneity. Significant heterogeneity was observed in the meta-analysis, which may not be fully explained by the variables included in the subgroup analyses, such as the study design, country, follow-up duration, or analytic models used. It could be hypothesized that other factors may have contributed to the heterogeneity, such as disease status and complications of the patients, as well as the methods and cutoffs for the diagnosis of myosteatosis. Moreover, we were unable to perform additional subgroup analysis according to the etiology of cirrhosis because none of the included studies reported stratified data according to the etiologies. The potential influence of the etiologies on the association between myosteatosis and the prognosis of patients with cirrhosis should be determined in future studies. Despite these factors, a meta-analysis using the random-effects model was performed to account for the observed heterogeneity. Besides, subgroup analysis based on the disease status (compensated vs. decompensated) or the clinical complication of cirrhosis is clinically important. However, since we did not have access to the individual patient data of the original studies included in the meta-analysis and as our meta-analysis was based on data at the study level rather than data at the individual-patient level, a subgroup analysis according to disease status or complications of cirrhosis could not be achieved. Moreover, univariate analysis was used in seven of the included studies, which may confound the results of the meta-analysis. However, subgroup analysis did not support that a difference in analytic model (univariate versus multivariate) may significantly affect the results. Additionally, our study solely encompassed observational studies, thus precluding the establishment of a causal relationship between myosteatosis and a compromised therapeutic response in individuals with liver cirrhosis. Consequently, it is crucial to ascertain whether interventions aimed at addressing myosteatosis could enhance the clinical outcomes of patients afflicted with liver cirrhosis. Finally, we only included full-length articles published in peer-reviewed journals in this meta-analysis, while excluding the gray literature, which may have led to the publication bias observed in this meta-analysis.

The findings of this meta-analysis suggest that incorporating myosteatosis assessment into risk stratification for patients with liver cirrhosis could offer significant clinical benefits. Implementing myosteatosis evaluations could help identify patients at higher risk of poor clinical outcomes, thereby enabling more targeted and personalized management strategies. For instance, clinicians could use myosteatosis assessment to guide decisions on more aggressive monitoring, early interventions, or personalized treatment plans, which would ultimately improve patient care and outcomes. To integrate myosteatosis assessment into clinical practice, healthcare providers might consider incorporating it into routine imaging studies or metabolic evaluations for patients with liver cirrhosis. Some practical steps could include standardizing diagnostic criteria for myosteatosis and establishing clear guidelines for their interpretation and use in clinical decision-making. Future research should focus on validating these findings in diverse patient populations and exploring the potential benefits of myosteatosis assessment in clinical trials. Studies should also aim to establish standardized protocols for assessing myosteatosis and investigate its impact on various treatment outcomes and strategies. Additionally, research into the underlying mechanisms linking myosteatosis to clinical outcomes could provide deeper insights and inform more effective management approaches. Finally, recent studies have suggested that the albumin–myosteatosis gauge could offer a more accurate prediction of outcomes compared to using either marker alone because albumin levels provide information on liver function and nutritional status, while myosteatosis indicates muscle quality. The prognostic value of myosteatosis combined with other indicators for patients with cirrhosis should also be explored in future studies [45].

## 5. Conclusions

In conclusion, the findings from the meta-analysis suggest that patients with liver cirrhosis and myosteatosis may experience a lower TFS compared to those without myosteatosis. However, it is important to note that further large-scale prospective studies are required to validate these results. Nevertheless, these findings provide support for the notion that myosteatosis could potentially serve as a prognostic indicator for patients with cirrhosis. Additional research is necessary to investigate whether improving myosteatosis could potentially lead to improved survival outcomes in this patient population.

## Supporting information

S1 Checklist PRISMA-2009 checklist. (DOC)

S1 Table Excluded studies and the reasons for exclusion. (DOC)

## Abbreviations

CT: computed tomography
TIPS: transjugular intrahepatic portosystemic shunt
TFS: transplant-free survival
HCC: hepatocellular carcinoma
NOS: Newcastle–Ottawa Scale
RRs: risk ratios
CIs: confidence intervals
SEs: standard errors
MELD: model for end-stage liver disease
HE: hepatic encephalopathy
