# Supplementary material for: Myosteatosis and the clinical outcomes of patients with liver cirrhosis: A meta-analysis
Source: PLoS One. 2024 Sep 12;19(9):e0310017. doi: 10.1371/journal.pone.0310017 (PMC11392268; doi:10.1371/journal.pone.0310017)
Supplement: S1 Table — (DOC) [file pone.0310017.s002.doc]

**S1 Table. Excluded studies and the reasons for exclusion**

| Study, yr | Justification | Citation |
| --- | --- | --- |
| Bhanji 2018 | Overlapped patients | Myosteatosis and sarcopenia are associated with hepatic encephalopathy in patients with cirrhosis. Hepatol Int. 2018;12(4):377-386. |
| Kitajima 2018 | Myosteatosis not evaluated as exposure | Supplementation with branched-chain amino acids ameliorates  hypoalbuminemia, prevents sarcopenia, and reduces fat  accumulation in the skeletal muscles of patients with liver  cirrhosis. J Gastroenterol. 2018;53(3):427-437. |
| Tachi 2018 | Outcome of interest not reported | Skeletal muscle fat deposition is associated with hepatocellular carcinoma development in patients with chronic liver disease. Nutrition. 2018:54:83-88. |
| Jahangiri 2019 | Myosteatosis not evaluated as exposure | Muscle Gain after Transjugular Intrahepatic Portosystemic Shunt Creation: Time Course and Prognostic Implications for Survival in Cirrhosis. J Vasc Interv Radiol. 2019;30(6):866-872.e4. |
| Bhanji 2019 | Outcome of interest not reported | Differing Impact of Sarcopenia and Frailty in Nonalcoholic Steatohepatitis and Alcoholic Liver Disease. Liver Transpl. 2019;25(1):14-24. |
| Hamaguchi 2020 | Patients after liver transplant | Including body composition in MELD scores improves mortality prediction among patients awaiting liver transplantation. Clin Nutr. 2020 Jun;39(6):1885-1892. |
| Czigany 2020 | Patients after liver transplant | Myosteatosis to predict inferior perioperative outcome in patients undergoing orthotopic liver transplantation. Am J Transplant. 2020;20(2):493-503. |
| Alexopoulos 2021 | Overlapped patients | Myostatin in combination with creatine phosphokinase or albumin may differentiate patients with cirrhosis and sarcopenia. Am J Physiol Gastrointest Liver Physiol. 2021;1;321(5):G543-G551. |
| Czigany 2021 | Patients after liver transplant | The role of recipient myosteatosis in graft and patient survival after deceased donor liver transplantation. J Cachexia Sarcopenia Muscle. 2021;12(2):358-367. |
| Bot 2021 | Not in patients with cirrhosis | Both muscle quantity and quality are predictors of waiting list mortality in patients with end-stage liver disease. Clin Nutr ESPEN. 2021:42:272-279. |
| Gioia 2021 | Outcome of interest not reported | The improvement in body composition including subcutaneous and visceral fat reduces ammonia and hepatic encephalopathy after transjugular intrahepatic portosystemic shunt. Liver Int. 2021;41(12):2965-2973. |
| Linge 2022 | Not in patients with cirrhosis | Adverse muscle composition is a significant risk factor for all-cause mortality in NAFLD. JHEP Rep. 2022; 24;5(3):100663. |
| Ishizu 2022 | Outcome of interest not reported | Factors associated with the progression of myosteatosis in patients with cirrhosis. Nutrition. 2022:103-104:111777. |
| Bot 2023 | Outcome of interest not reported | Skeletal muscle mass in patients with end-stage liver disease: Not only muscle size but especially muscle quality matters in relation to physical fitness. Clin Nutr ESPEN. 2023:55:407-413. |
| Praktiknjo 2023 | Not in patients with cirrhosis | Myosteatosis independently predicts transplant-free survival in patients with primary sclerosing cholangitis. Dig Liver Dis. 2023;55(11):1543-1547. |
| Petric 2023 | Patients after liver transplant | Radiological assessment of skeletal muscle index and myosteatosis and their impact postoperative outcomes after liver transplantation. Radiol Oncol. 2023;21;57(2):168-177. |
| Thuluvath 2024 | Outcome of interest not reported | Utilizing a novel MRI technique to identify adverse muscle composition in  end-stage liver disease: A pilot study. Ann Hepatol. 2024;29(4):101508. |
| Zeng 2024 | Not in patients with cirrhosis | Sarcopenia is associated with short- and long-term mortality in patients with acute-on-chronic liver failure. J Cachexia Sarcopenia Muscle. 2024;15(4):1473-1482. |
| Geng 2024 | Not in patients with cirrhosis | Association of myosteatosis with short‑term outcomes in patients with acute‑on‑chronic liver failure. Sci Rep. 2024;13;14(1):13609. |
